# Platelet-Rich Plasma in Episiotomy Repair: A Critical Appraisal of a Sparse and Partly Non-Indexed Evidence Base and Rationale for a Randomized Controlled Trial

**DOI:** 10.3390/life16081339

**Published:** 2026-08-15

**Authors:** Dragos Brezeanu, Ana-Maria Brezeanu, Traian-Virgiliu Surdu, Monica Surdu, Vlad Tica

**Affiliations:** 1 Faculty of Medicine, Ovidius University, 900527 Constanta, Romania; brezeanudragos@gmail.com (D.B.); traian@surdu.ro (T.-V.S.); monica@surdu.ro (M.S.); vtica@eeirh.org (V.T.); 2 County Clinical Emergency Hospital “St. Apostle Andrew”, 900591 Constanta, Romania; 3 Techirghiol Balneal and Rehabilitation Sanatorium, 906100 Constanta, Romania; 4 Romanian Academy of Scientists, 050045 Bucharest, Romania

**Keywords:** platelet-rich plasma, episiotomy, perineal wound healing, postpartum recovery, regenerative medicine, randomized controlled trial

## Abstract

Background: Episiotomy remains one of the most common obstetric procedures worldwide, and suboptimal healing of the perineal wound continues to cause pain, dehiscence, and long-term impairment of postpartum quality of life. Obstetric anal sphincter injury (OASI) carries the greatest risk of severe wound morbidity among perineal trauma types and is managed with prophylactic antibiotics and adjunctive measures; episiotomy, by contrast, is repaired without a comparable prophylactic package and is far more frequent, so that even a modest per-case complication rate carries a substantial absolute burden. Platelet-rich plasma (PRP), an autologous concentrate rich in growth factors, has demonstrated favourable effects on wound healing and scar quality across several surgical contexts, including caesarean section, raising the question of whether it could similarly benefit episiotomy repair. Methods: We conducted a structured, date-stamped search of PubMed, Scopus and Google Scholar, together with two trial registries (ClinicalTrials.gov, WHO ICTRP), forward and backward citation tracking, and reference-list screening of relevant reviews (final search 24 July 2026), to identify clinical studies of PRP for obstetric episiotomy or intrapartum perineal wound healing. Results: Five primary clinical studies and one systematic review were identified. One single-centre randomized controlled trial of 200 primiparous women, published in a regionally indexed journal covered by neither PubMed nor Scopus, evaluated PRP specifically for episiotomy wound healing and reported significantly lower REEDA, Vancouver and pain scores in the PRP arm; it was not prospectively registered, reported neither a sample-size calculation nor blinded outcome assessment, lost 12% of participants to follow-up, and contains internal inconsistencies in the reported data. The remaining evidence comprises one randomized trial of PRP for postpartum levator ani muscle recovery (a distinct target, with a null result), one non-randomized comparative study in grade III–IV intrapartum perineal laceration repair, two case reports, and one broad systematic review of PRP in pelvic floor disorders in which episiotomy was not analyzed as a distinct entity. Conclusions: PRP for episiotomy healing is therefore not wholly untested, but the single existing randomized trial is small, methodologically limited, not independently replicated, and effectively invisible to conventional database searching. We describe this evidence base and introduce PRP-EpiHeal (ClinicalTrials.gov ID: NCT07669285), designed to provide the first prospectively registered, adequately powered and assessor-blinded randomized evidence on this question, with REEDA scale assessment at six weeks postpartum as the primary outcome.

## 1. Introduction

Episiotomy remains one of the most frequently performed obstetric procedures worldwide, despite a sustained, decades-long shift toward more selective and restrictive use [1]. The World Health Organization, in its technical guidance on care in normal birth, found no evidence supporting the liberal use of episiotomy and noted that a rate in the region of 10% for normal vaginal deliveries had been achieved without harm to mother or infant [2]; several recent institutional and population-based studies report rates substantially above this figure [3,4]. Reported prevalence still ranges widely across settings, from below 10% in countries following a sustained restrictive policy to rates exceeding 60% in several European countries, including Romania, reflecting persistent heterogeneity in obstetric practice [5]. Regardless of the exact local rate, episiotomy continues to represent one of the most common surgical interventions performed on women globally, and its wound-healing outcomes therefore carry substantial public health relevance. In a recent multicentre UK prospective cohort of over 2000 women with childbirth-related perineal trauma, wound infection was documented in 9.5% of those who had undergone episiotomy [6]. This figure should be read in its clinical context. Obstetric anal sphincter injury (OASI) is the perineal trauma category associated with the greatest risk of severe wound complications, and its management routinely incorporates prophylactic broad-spectrum antibiotics, stool softeners and structured follow-up, which attenuate the observed infection rate. Episiotomy, by contrast, is repaired without any comparable prophylactic package, and is one to two orders of magnitude more frequent. The clinical relevance of the episiotomy wound therefore derives from the combination of a non-trivial per-case complication rate and a very large denominator, not from any claim that it is the most severe form of perineal trauma.

While most episiotomy wounds heal without incident, a non-trivial proportion of women experience delayed healing, wound dehiscence, infection, hematoma, or persistent discharge, with reported infection rates of approximately 5–10% in large recent cohorts [6]. Beyond the immediate postpartum period, these complications are associated with chronic perineal pain, dyspareunia, and impaired sexual quality of life, with consequences that can extend for months after delivery and affect a woman’s ability to care for her newborn, resume daily activities, and breastfeed comfortably [7,8]. Despite this burden, current management of the episiotomy wound remains largely supportive in nature: local hygiene measures, analgesia, and expectant observation, with no widely adopted intervention specifically designed to actively enhance the biological process of perineal wound repair [7,8].

Platelet-rich plasma (PRP) has emerged over the past two decades as a regenerative strategy across multiple surgical and musculoskeletal disciplines. Prepared by centrifugation of a patient’s own peripheral blood, PRP yields an autologous concentrate enriched in platelets and the growth factors they release upon activation, including platelet-derived growth factor, transforming growth factor-beta, and vascular endothelial growth factor which are thought to accelerate angiogenesis, modulate inflammation, and promote organized tissue repair [9,10]. Since its early use in cardiac surgery in the late 1980s, PRP has been adopted in orthopedics, sports medicine, dermatology, and plastic and reconstructive surgery, with encouraging results in wound healing and scar reduction [11,12]. Because PRP is autologous, it carries a comparatively low risk of allergic reaction or transmissible infection relative to allogeneic or synthetic alternatives [12,13].

Within obstetrics specifically, PRP has previously been investigated in the context of caesarean section wound healing, where it was infiltrated intraoperatively at both the uterine incision (hysterotomy) margins and the subcutaneous skin layer; favourable effects were reported on skin-level scar quality in a single-centre randomized controlled pilot trial and a prospective observational study, although healing at the level of the myometrium itself was not separately assessed [13,14]. Consistent benefits have since been reported by other groups, further supporting a role for PRP in caesarean wound healing [15]. In parallel, we have also studied a different regenerative approach, topical lactic acid, specifically for episiotomy wound healing, through a randomized clinical trial of intravaginal lactic acid gel [16]. This dual line of work prompted us to examine the literature on PRP in episiotomy healing directly. The exercise proved instructive in an unexpected way: the answer returned by a conventional search of the major international databases differs materially from the answer returned once regionally indexed journals, trial registries and citation tracking are added. Any assessment of what is known about PRP in episiotomy repair therefore depends critically on how the literature is searched, and this observation motivates both the methodological approach and the framing of the present communication.

This asymmetry is not necessarily trivial to resolve by extrapolation. The caesarean incision and the episiotomy wound differ substantially in several respects relevant to healing biology: tissue composition and vascularity, degree and type of bacterial exposure (the episiotomy wound heals in immediate proximity to the vaginal and perianal microbiota), and the mechanical environment during the early postpartum period, where the perineum is subject to sitting, walking, and continuous activity that the caesarean incision is not [13,14,15,16]. These differences mean that favourable results obtained for PRP in caesarean wound healing cannot be assumed to generalize to the episiotomy wound without dedicated clinical evaluation.

It is also worth clarifying the scope of perineal trauma addressed here. Episiotomy is a deliberate surgical incision and constitutes only one form of perineal trauma; spontaneous intrapartum perineal lacerations (tears), which are not eliminated by a restrictive episiotomy policy, represent a related but distinct category. Episiotomy itself is not a single, uniform procedure: mediolateral and midline techniques carry different risks of extension toward the anal sphincter, and the broader intrapartum context, spontaneous versus operative (forceps- or vacuum-assisted) vaginal delivery, together with maternal, fetal, and constitutional factors, further shapes both the occurrence and the healing trajectory of perineal trauma more generally. The present communication, and the PRP-EpiHeal trial it introduces, focus specifically on the episiotomy wound rather than on spontaneous perineal lacerations or obstetric anal sphincter injury as separate entities; readers should bear this distinction in mind when considering the boundaries of the evidence described below.

In this communication we report a structured, reproducible search undertaken to determine whether and how PRP has been evaluated for episiotomy or intrapartum perineal wound healing; we appraise critically the small number of studies identified, including one randomized trial that is not retrievable through PubMed or Scopus; and we describe PRP-EpiHeal (ClinicalTrials.gov ID: NCT07669285), a two-arm randomized controlled trial designed by our group to provide prospectively registered, adequately powered and assessor-blinded evidence on this question.

## 2. Materials and Methods

A structured literature search was conducted to identify clinical studies evaluating platelet-rich plasma (PRP) for obstetric episiotomy or intrapartum perineal wound healing following vaginal delivery (spontaneous or operative), as distinct from gynecological perineal procedures. The search was designed to be reproducible; the full database-specific strategies, dates and yields are reported below. The final search was executed on 24 July 2026 and covers records available up to that date.

Databases and registries. PubMed (MEDLINE), Scopus and Google Scholar (first 200 hits, relevance-ranked) were searched, together with ClinicalTrials.gov and the WHO International Clinical Trials Registry Platform (ICTRP) for ongoing or unpublished trials. Grey literature was addressed by screening conference abstracts retrieved through Google Scholar and by examining the reference lists of all included records and of the most recent systematic reviews in the field, followed by forward citation tracking of each included record in Google Scholar and Scopus.

Search strings: PubMed: (“Platelet-Rich Plasma” [Mesh] OR PRP[tiab] OR “platelet rich plasma” [tiab]) AND (“Episiotomy” [Mesh] OR “Obstetric Surgical Procedures” [Mesh] OR “Delivery, Obstetric” [Mesh] OR “Birth Injuries” [Mesh] OR episiotomy[tiab] OR “perineal wound” [tiab] OR “perineal trauma” [tiab] OR “perineal tear” [tiab] OR “perineal laceration” [tiab]). Scopus: TITLE-ABS-KEY ((“platelet-rich plasma” OR PRP) AND (episiotomy OR “obstetric surgical procedures” OR “delivery, obstetric” OR “birth injuries” OR “perineal wound” OR “perineal trauma” OR “perineal tear” OR “perineal laceration”)). Google Scholar: (“platelet-rich plasma” OR PRP) AND (episiotomy OR “perineal wound” OR “perineal tear”). No restriction on publication date or study design was applied at the search stage. No language restriction was applied at the search stage, and non-English records were assessed with the assistance of published translations where available.

Records were included if they reported original clinical data (randomized controlled trials, non-randomized comparative studies, case series, or case reports) on PRP application for obstetric episiotomy or intrapartum perineal wound repair after vaginal delivery. Records were excluded if they addressed non-obstetric (gynecological) perineal or vaginal procedures; pelvic floor conditions without a distinct episiotomy or intrapartum perineal-trauma component (e.g., pelvic organ prolapse, stress urinary incontinence, vulvovaginal atrophy, lichen sclerosus); or non-clinical (in vitro or animal) studies. Systematic reviews were retained separately as secondary syntheses rather than as primary evidence.

Records were exported to a reference manager and de-duplicated automatically by DOI and title, followed by manual verification. Titles and abstracts were screened independently by two authors (D.B., A.-M.B.); full texts of potentially eligible records were retrieved and assessed against the criteria above by the same two authors, with disagreements resolved by discussion and, where necessary, adjudication by a third author (V.T.). Data were extracted into a standardized form covering design, setting, population and sample size, PRP preparation, comparator, outcome instruments, follow-up duration, and principal findings.

**Figure 1:**
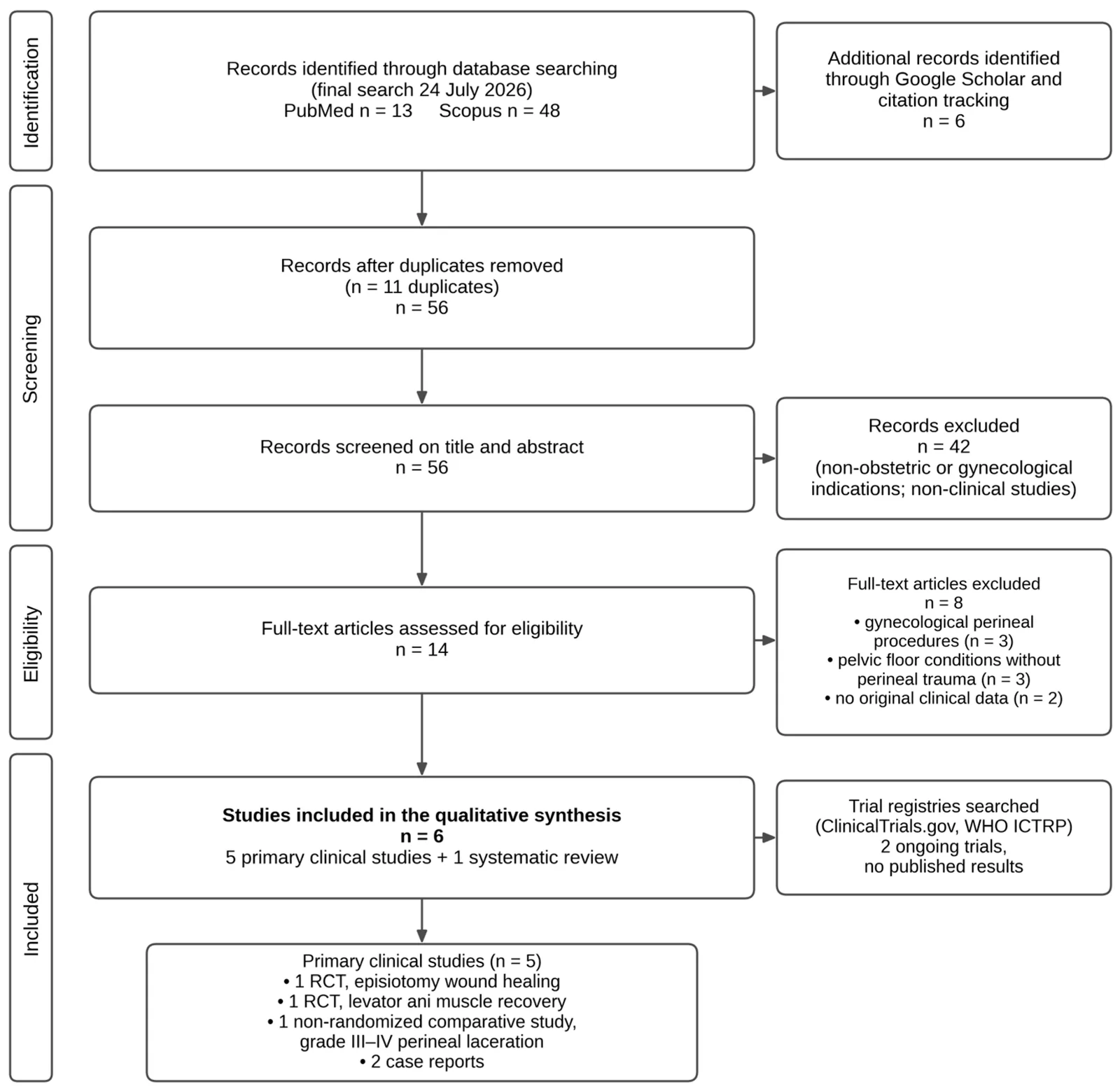
Literature search flow for the identification of clinical evidence on platelet-rich plasma (PRP) in obstetric episiotomy or intrapartum perineal wound healing following vaginal delivery [17,18,19,20,21,22].

The PubMed query returned 13 records and the Scopus query 48 records; Google Scholar and citation tracking contributed a further 6 unique records not retrievable in either database, giving 67 records in total. After removal of 11 duplicates, 56 records were screened on title and abstract, of which 42 were excluded as addressing non-obstetric or gynecological indications, pelvic floor conditions without a distinct perineal-trauma component, or non-clinical work. Fourteen full-text articles were assessed for eligibility and 8 were excluded, leaving 5 primary clinical studies and 1 systematic review. The registry search identified 2 ongoing trials with no published results. The stages of the search and the records retained at each step are summarized in Figure 1.

Given the focused, hypothesis-generating purpose of this communication, the search was not registered as a systematic review protocol and does not claim full PRISMA compliance. It nevertheless follows PRISMA principles for reproducibility, explicit and complete search strings, a stated search date, duplicate screening, documented eligibility criteria, and a flow diagram, so that the retrieval can be independently verified or updated.

## 3. Results

The structured search identified five primary clinical studies and one systematic review. Only one of these evaluated PRP specifically for episiotomy wound healing in a randomized design: a single-centre trial conducted at two Egyptian hospitals between May and December 2021 and published in 2023 in a regionally indexed journal that is covered by neither PubMed nor Scopus [17]. Two hundred primiparous women expected to undergo episiotomy were randomized 1:1 by computer-generated sequence with sealed envelopes to subcutaneous injection of autologous PRP into the wound before skin closure, or to saline cleansing alone; 176 women were analyzed. REEDA, Vancouver Scar Scale and visual analogue pain scores were assessed on day one and at one, two and four weeks. The authors reported significantly lower REEDA and Vancouver scores in the PRP arm at one and two weeks, lower pain scores from week one onwards, and no cases of wound gaping in the PRP arm compared with seven among controls.

That trial is directly on point, and its existence must be acknowledged; it is, however, subject to important methodological limitations, which are appraised in Section 4.2. A second randomized trial applied PRP during postpartum perineorrhaphy but targeted recovery of the levator ani muscle rather than wound healing, assessing levator hiatal area and pelvic floor muscle strength; of 240 women enrolled, 58 completed follow-ups, and no significant between-group difference was demonstrated [18].

The remaining primary evidence does not address the uncomplicated episiotomy wound. A non-randomized, post-test-only comparative study evaluated PRP on wound healing, anal continence and sphincter tone after repair of grade III–IV intrapartum perineal laceration [19]. Two case reports describe PRP used as rescue treatment rather than as primary prophylaxis: one in a woman with episiotomy dehiscence following vacuum-assisted delivery, in whom serial autologous PRP injections were followed by complete healing [20], and one in a woman with chronic pain after a deep episiotomy with an associated grade II perineal laceration [21].

The most comprehensive synthesis remains a systematic review of PRP in pelvic floor disorders, which screened 644 articles across six databases and identified 15 eligible studies involving 600 women treated with PRP for a range of pelvic floor conditions [22]. Intrapartum perineal trauma was among the conditions considered, alongside pelvic organ prolapse, stress urinary incontinence, female sexual dysfunction, vulvovaginal atrophy, and vesicovaginal fistula, but episiotomy wound healing was not analyzed as a distinct clinical entity, and the review did not retrieve the randomized trial described above.

Taken together, the identified evidence is summarized in Table 1. It is sparse, methodologically heterogeneous, concentrated in journals with limited international indexing, and—with the single exception noted above—not directed at the episiotomy wound as a distinct entity.

## 4. Discussion

The principal finding of this structured search is not that PRP has never been tested in episiotomy repair, but that it has been tested once, in a small trial that is effectively invisible to the standard literature. Our own initial assumption, shared as far as we can determine, by the most recent systematic review in this field, that no randomized evidence existed proved to be an artefact of database coverage rather than a fact about the world [22]. We consider this worth stating explicitly, because it bears directly on how evidence gaps in obstetrics are identified and how confidently they can be asserted.

### 4.1. What the Identified Evidence Does and Does Not Show

The evidence summarized in Table 1 supports three statements. First, PRP has been applied to the episiotomy wound in a randomized comparison, and the reported direction of effect was favourable across healing, pain and dehiscence outcomes [17]. Second, that comparison stands alone: it has not been replicated, and the remaining primary studies address either a different anatomical target [18], a different clinical entity [19], or established complications treated as salvage rather than prevented at primary repair [20,21]. Third, the only systematic synthesis available did not analyze episiotomy separately and did not retrieve the randomized trial, so it cannot be relied upon as a summary of the evidence for this indication [22].

### 4.2. Critical Appraisal of the Existing Randomized Trial

The trial by Ali and colleagues is the only randomized comparison of PRP versus standard care for episiotomy wound healing that we could identify [17]. Its design is appropriate in principle: a computer-generated randomization sequence with block sizes of six, concealed in sequentially numbered sealed envelopes; an adequately defined primiparous population; exclusion of conditions known to impair healing; a standardized PRP preparation protocol with reported centrifugation parameters and calcium chloride activation; and validated outcome instruments (REEDA, Vancouver Scar Scale, VAS) [17].

Several features nevertheless limit the strength of the inference that can be drawn. The trial was not prospectively registered, so the pre-specified primary outcome and analysis plan cannot be verified. No sample-size calculation is reported, and the choice of 200 participants is not justified. There is no statement that outcome assessors were blinded to allocation; given that REEDA and Vancouver scores are observer-rated and that the control condition was saline cleansing rather than a sham injection, unblinded assessment is a plausible source of bias in the direction of the reported effect. Twenty-four of 200 randomized women (12%) were lost to follow-up, and analysis was restricted to completers rather than performed by intention to treat. Ordinal composite scores were compared using parametric t-tests. The reported REEDA values at four weeks are numerically identical in the two arms yet accompanied by a *p*-value of 0.0001, and the day-one VAS values are numerically higher in the PRP arm than in controls while being presented as favouring PRP; these internal inconsistencies are not addressed in the published report. Finally, follow-up ended at four weeks, before the six-week window in which perineal healing is conventionally assessed and in which late dehiscence and infection may still declare themselves.

None of this establishes that the reported effect is spurious. The direction and magnitude of the findings are biologically coherent and consistent with the caesarean literature [13,14,15]. The appropriate conclusion is narrower and more useful: a single small trial provides a promising but fragile signal that has not been independently replicated, has not been subject to prospective registration or blinded outcome assessment, and would not, on its own, support a change in clinical practice. The relevant gap is therefore not the absence of any randomized evidence, but the absence of confirmatory randomized evidence of adequate methodological quality.

### 4.3. Why Extrapolation from Adjacent Evidence Remains Insufficient

Episiotomy and grade III–IV intrapartum perineal laceration, although anatomically adjacent, differ substantially in clinical context, intervention timing, and outcome relevance. Episiotomy repair is performed in the immediate aftermath of an otherwise uncomplicated vaginal delivery, in a population that is overwhelmingly healthy, and the clinically meaningful endpoints—wound redness, edema, ecchymosis, discharge, and approximation of the wound edges, alongside pain and resumption of normal activity—are distinct from the continence- and sphincter-function-oriented endpoints relevant to severe perineal trauma. Evidence generated in one context cannot be assumed to transfer to the other. The same applies to the randomized evidence on levator ani muscle recovery [18]: a null result for restoring muscle bulk and strength months after delivery carries little information about epithelial and connective-tissue healing of a surgical incision over the first six weeks. Similarly, the two case reports [20,21] describe PRP deployed as salvage therapy for an established complication, which is a different clinical question from prophylactic application at the time of primary repair.

The case for testing PRP in this setting is unlikely to founder on biological plausibility. PRP has previously shown favourable effects on caesarean scar healing [13,14], and a topical lactic acid gel has separately been validated for episiotomy wound healing through a randomized trial [16]; taken together, this body of work indicates that regenerative strategies are being actively investigated for each of these two obstetric wound types individually, and the underlying biological rationale—platelet-derived growth factors promoting angiogenesis, modulating local inflammation, and supporting organized tissue repair—applies in principle to both wound types [9,10]. Our group’s prior work in this area, and our role as investigators in the trial introduced below, are disclosed transparently in Section 4.5 and in the Conflicts of Interest statement.

### 4.4. PRP-EpiHeal: Design and Rationale

PRP-EpiHeal (ClinicalTrials.gov ID: NCT07669285) is a two-arm, parallel-group, open-label randomized controlled trial with blinded outcome assessment, conducted at two centres. Eligible participants are women aged 18 years or older undergoing vaginal delivery at term with mediolateral episiotomy, who provide written informed consent. Exclusion criteria include coagulopathy or anticoagulant therapy, thrombocytopenia, active local or systemic infection, immunosuppression, poorly controlled diabetes, extension to third- or fourth-degree laceration, and any condition precluding follow-up. Full eligibility criteria are available in the trial registration.

Randomization and blinding. Participants are allocated 1:1 by a computer-generated sequence with variable block sizes, stratified by centre, concealed through a central web-based system accessed after delivery and after the decision to perform episiotomy has been taken. Because PRP preparation requires venepuncture and centrifugation, the delivering clinician cannot be blinded; the primary outcome is therefore assessed by a trained clinician independent of the delivery team, blinded to allocation, and without access to the delivery record. Participants are informed of the study design but are not reminded of their allocation at follow-up visits, and statistical analysis is performed on de-identified, allocation-masked datasets.

PRP preparation and administration. Under aseptic conditions, autologous venous blood is collected into sodium-citrate tubes immediately after delivery and processed by a standardized two-step centrifugation protocol; the platelet-rich fraction is separated without disturbing the buffy coat, and the platelet concentration of the final product is quantified for each participant, in accordance with published recommendations for standardized PRP reporting [23,24]. The concentrate is infiltrated into the wound margins of the episiotomy at the subcutaneous and muscular levels immediately before skin closure, using a single pre-specified volume. Collection volume, centrifugation parameters, activation method, final platelet concentration and injected volume are pre-specified in the trial protocol and will be reported in full alongside the results.

Standardization of co-interventions. Episiotomy is performed mediolaterally and repaired by continuous non-locking suture with polyglactin 910 according to a single written unit protocol in both arms. Antibiotic prophylaxis, analgesia, and perineal hygiene advice follow the same standardized regimen in both arms, and any deviation is recorded.

### 4.5. Outcomes, Sample Size and Statistical Plan

The primary outcome is the total REEDA score [25,26] at six weeks postpartum, assessed by the blinded outcome assessor. The six-week timepoint was chosen because it corresponds to the conventional end of the puerperium and to the follow-up window used by the largest contemporary cohort of perineal wound complications [6], and because it captures late-declaring dehiscence and infection that the four-week endpoint of the existing trial may miss [17]. Secondary outcomes include longitudinal visual analogue scale pain scores [27], wound dehiscence, wound infection, need for secondary suturing, and maternal satisfaction. Assuming a between-group difference of 0.5 points in mean REEDA score with a standard deviation of 1.1 (standardized effect size 0.45), 78 participants per arm provide 80% power at a two-sided alpha of 0.05; allowing for up to 20% attrition, the recruitment target is 200 participants. Analysis will follow the intention-to-treat principle, with between-group comparison of the primary outcome by Mann–Whitney U test and a supportive analysis of covariance adjusted for centre and maternal age; a per-protocol analysis is pre-specified as a sensitivity analysis. The full statistical analysis plan is finalized before database lock.

Safety, ethics and consent. Adverse events are recorded systematically at each visit, with particular attention to injection-site reactions, hematoma, infection and hospital readmission. The protocol was approved by the institutional ethics committees of both participating centres, and written informed consent is obtained antenatally or in early labour, before the decision to perform episiotomy, so that consent is not sought under the time pressure of the second stage. The trial protocol was developed in accordance with the SPIRIT recommendations for protocol content, and results will be reported according to the CONSORT statement [28].

Because PRP is autologous and requires no specialized equipment beyond a centrifuge, it could in principle translate readily into routine intrapartum care if, and only if, the trial demonstrates both efficacy and an acceptable safety profile; we make no claim of clinical benefit ahead of that evidence. We present the rationale for PRP-EpiHeal with appropriate caution regarding the direction of its eventual results. Biological plausibility, the favourable signal from caesarean wound healing, and the single existing episiotomy trial support the hypothesis that PRP may benefit episiotomy repair, but they do not establish it; the null result of the levator ani trial is a reminder that PRP does not benefit every postpartum tissue target, and the perineal wound environment differs sufficiently from the caesarean incision that a null result remains a credible outcome [18]. It is precisely because the answer is not yet secure despite being biologically plausible and clinically important that a rigorously conducted randomized controlled trial, rather than continued extrapolation from adjacent evidence, is the appropriate next step for this field.

### 4.6. Limitations

Several limitations should be acknowledged. First, although the search reported here is explicit, dated and reproducible, it is not a registered systematic review and does not claim full PRISMA compliance; formal risk-of-bias assessment using a validated instrument was not undertaken, and the appraisal in Section 4.2 is narrative. Our own experience in this review—identifying a directly relevant randomized trial only after extending the search beyond PubMed and Scopus—illustrates that further studies published in regionally indexed or non-English sources may still have escaped detection.

Second, this is a perspective and hypothesis-generating communication rather than an original data report; it does not itself present clinical outcomes, and the trial it introduces, PRP-EpiHeal, is currently in the pre-recruitment phase. The arguments advanced here therefore rest on biological plausibility, on extrapolation from adjacent indications, and on a single small, randomized trial of limited methodological quality, and must be interpreted as a rationale for investigation rather than as evidence of efficacy.

Third, as members of the group that designed PRP-EpiHeal and that has previously published on both PRP in caesarean healing and lactic acid in episiotomy healing, we hold an inherent interest in this research area. We have sought to present the evidence gap and the trial’s rationale descriptively and transparently, including explicit acknowledgement that a null result is a credible outcome, but readers should weigh this perspective accordingly.

Fourth, the primary endpoint of PRP-EpiHeal is assessed at six weeks postpartum, and the trial does not currently include longer-term follow-up as a tertiary or exploratory outcome. Because chronic perineal pain and dyspareunia can persist for months, a six-week endpoint may not capture the full clinically meaningful effect of the intervention even if the primary result is positive; we note this as a limitation of the current design and a priority for future extension or follow-up studies.

### 4.7. Future Directions

The most immediate priority is the completion of adequately powered, prospectively registered randomized controlled trials with blinded outcome assessment, evaluating PRP specifically for episiotomy wound healing and using validated, clinically meaningful endpoints such as the REEDA score, standardized pain scales, and objective complication rates. PRP-EpiHeal is intended as a step in this direction, but two-centre trials will need to be complemented by larger multicenter studies to establish generalizability across populations, delivery practices, and episiotomy techniques (midline versus mediolateral). Independent replication of the findings reported by Ali and colleagues [17], in a registered and assessor-blinded design, is a specific and tractable objective.

Beyond efficacy, future work should address the standardization of PRP preparation, platelet concentration, activation method, injection volume, and timing relative to suture closure, since heterogeneity in these parameters has historically limited the comparability and reproducibility of PRP research across disciplines [23,24]. Harmonized reporting standards would substantially strengthen any future systematic review or meta-analysis in this area, which at present would rest on a single randomized comparison. Improving the indexing and discoverability of regionally published obstetric trials is a related and equally practical priority.

Finally, should PRP demonstrate benefit in episiotomy healing, subsequent research should examine longer-term outcomes that matter to women beyond the six-week postpartum window, including scar quality, chronic perineal pain, and sexual function, as well as health-economic evaluations to determine whether widespread application is cost-effective at scale [29]. Recently developed postpartum patient-reported outcome instruments may provide an additional framework for evaluating the long-term impact of regenerative interventions on women’s postpartum recovery and sexual well-being [29].

## 5. Conclusions

PRP has a strong biological rationale in perineal wound repair and is well established in adjacent surgical fields, yet the evidence base for its use in episiotomy repair consists of a single small, randomized trial, published outside the major citation databases and not independently replicated, together with a handful of non-randomized reports addressing different clinical entities. This is a real and clinically consequential evidentiary weakness concerning one of the most common surgical procedures performed on women worldwide, and it is compounded by the fact that the only directly relevant trial is difficult to retrieve by conventional means. The absence of confirmatory evidence should not be mistaken for evidence of absence of benefit; rather, it identifies a question that is biologically plausible, clinically important, and not yet securely answered. PRP-EpiHeal (ClinicalTrials.gov ID: NCT07669285) has been designed and prospectively registered to provide adequately powered, assessor-blinded randomized evidence on this question. We hope this communication both encourages rigorous investigation of regenerative approaches to perineal wound healing and serves as a reminder that claims about the absence of evidence require a search strategy capable of supporting them.

## Figures and Tables

**Table 1:** Summary of the available clinical evidence on platelet-rich plasma (PRP) in intrapartum perineal trauma and episiotomy-related conditions.

Study	Design	Population (*n*=)	Main Findings	Relevance to Episiotomy Healing
Ali, 2023 [17]	Randomized controlled trial	Primiparous women undergoing episiotomy (*n* = 200 randomized; 176 analyzed)	Lower REEDA, Vancouver and VAS scores in the PRP arm at 1–2 weeks; no wound gaping vs. 7 in controls	Direct—but unregistered, no sample-size calculation, no blinded assessment, internal data inconsistencies
Moegni, 2022 [18]	Randomized controlled trial	Primiparous women after vaginal delivery (*n* = 240 enrolled; 58 analyzed)	No difference in levator hiatal area or pelvic floor muscle strength	Different target: muscle recovery, not wound healing
Kurniawati, 2026 [19]	Non-randomized, post-test-only comparative study	Women after repair of grade III–IV intrapartum perineal laceration (*n* = 30)	Improved wound healing and continence outcomes	Different clinical entity
Akhoundova, 2022 [20]	Case report	One woman with episiotomy dehiscence (*n* = 1)	Complete healing after serial autologous PRP injections	Anecdotal; rescue treatment of an established complication
Kabakçı, 2021 [21]	Case report	One woman with chronic pain after deep episiotomy with grade II laceration (*n* = 1)	Symptomatic improvement	Anecdotal; chronic pain, not acute wound healing
Kurniawati, 2023 [22]	Systematic review	Pelvic floor disorders (15 studies; *n* = 600 women)	Heterogeneous PRP outcomes; episiotomy not analyzed separately	Synthesis, not primary evidence

Note: the final row summarizes a systematic review, itself a synthesis of heterogeneous pelvic-floor indications rather than a primary data source; the preceding rows are primary clinical studies. “Relevance to episiotomy healing” reflects our assessment of applicability, not a formal measure of evidence quality. PRP, platelet-rich plasma; REEDA, Redness, Edema, Ecchymosis, Discharge, Approximation; VAS, visual analogue scale.

## Data Availability

No new data were created or analyzed in this study. Data sharing is not applicable to this article. The literature search underlying this communication was based on publicly available, published sources; the complete search strings and the search date are reported in Section 2 and permit independent replication. Details of the trial discussed (PRP-EpiHeal) are publicly available on ClinicalTrials.gov (identifier NCT07669285).

## Abbreviations

The following abbreviations are used in this manuscript:

WHO	World Health Organisation
PRP	Platelet-Rich Plasma
RCT	Randomized Controlled Trial
REEDA	Redness, Edema, Ecchymosis, Discharge, Approximation
VAS	Visual Analogue Scale
SR	Systematic Review
SUI	Stress Urinary Incontinence
POP	Pelvic Organ Prolapse
OASI	Obstetric Anal Sphincter Injury
ICTRP	International Clinical Trials Registry Platform
SPIRIT	Standard Protocol Items: Recommendations for Interventional Trials
CONSORT	Consolidated Standards of Reporting Trials
VSS	Vancouver Scar Scale
